# Quantitative measurements of PFAS at femtomole concentrations *via* integrated SERS and single photon detection methods

**DOI:** 10.1039/d5ra05114b

**Published:** 2025-10-15

**Authors:** Tianhang Huo, Yehong Li, Santosh Kumar, Silvana Andreescu, Yu-Ping Huang, Henry Du

**Affiliations:** a Department of Chemical Engineering and Materials Science, Stevens Institute of Technology 1 Castle Point Terrace Hoboken 07310 NJ USA thuo@stevens.edu; b Department of Physics, Stevens Institute of Technology 1 Castle Point Terrace Hoboken 07310 NJ USA; c Chemistry & Biochemistry Department, Clarkson University 8 Clarkson Ave Potsdam 13699 NY USA

## Abstract

Per- and polyfluoroalkyl substances (PFAS) pose significant environmental and health concerns, necessitating their efficient and accurate identification to facilitate their eventual mitigation from the environment. Surface-enhanced Raman spectroscopy (SERS) enables highly sensitive and precise molecular identification, but trace-level detection of chemicals and fluorescence interference remain significant challenges. Here, we present a uniform 3D AgNP@Si substrate for SERS, leveraging photon counting to achieve susceptible and low-fluorescence detection. This approach enables the detection of PFAS, including perfluorooctanoic acid (PFOA) and perfluorooctane sulfonate (PFOS), at concentrations as low as 10^−15^ M, with Rhodamine 6G (R6G) used as a model analyte. Additionally, the quantitative analysis demonstrated a strong logarithmic relationship between Raman intensity and analyte concentration, with high correlation coefficients (*R*^2^ = 0.98 for R6G and 0.97 for PFOA and PFOS). This pioneering approach offers a promising alternative to current analytical techniques for monitoring PFAS and other contaminants in the environment.

## Abstract 2

Sub-ppt detection of PFAS is enabled by combining multilayer AgNPs SERS substrates with photon-counting detection.

## Graphical abstract

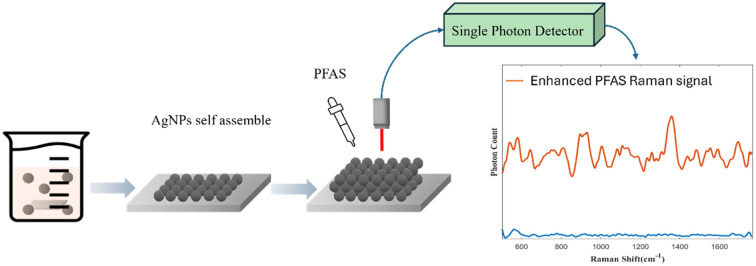

## 1 Introduction

PFAS are synthetic compounds containing perfluorinated methyl (–CF3) and methylene (–CF2) groups. These compounds, also labelled “forever chemicals”, are known for their chemical stability, environmental persistence, and resistance to degradation. Widely used for over eight decades in industrial and consumer goods such as firefighting foams, food packaging, water-repellent fabrics, and non-stick cookware, their ubiquitous presence has led to environmental contamination and human exposure through products, soil, air, and water.^1–3^ PFAS accumulate in the body over time and have been linked to health risks, including developmental issues, liver damage, immune system impairment, thyroid disruption, and cancers. Pollution of PFAS in the environment and its adverse effects have emerged as one of the most alarming public health concerns.^4–9^ These concerns promoted the EPA to establish a safe drinking water standard of maximum 4 ppt for highly hazardous PFAS in April 2024.^10^ This standard is informed, to a significant degree, by the limit of detection of PFAS in water by currently available state-of-the-art analytical techniques.

Liquid chromatography-mass spectrometry (LC-MS/MS) is the gold standard for detection and qualification of PFAS at ppt levels. LC-MS/MS is mainly a laboratory-based and sophisticated analytical tool. They often involve complex sample preparation procedures and well-trained instrument operators. As a result, the operation cost is high and analysis throughput is limited, making LC-MS/MS cost-prohibitive and time-inefficient to aid in assessment of wide-spread PFAS contaminations at scale.^11–14^ Any alternative technique that can achieve and even surpass the capabilities of LC-MS/MS for detection of PFAS without its excessive cost and time constraints will great enhance our ability to identify and ultimately mitigate PFAS contaminations in the environment. Detection of PFAS at ppt to sub-ppt levels by modern analytical methods such as electrochemical measurements and laser spectroscopies is extremely challenging, however this difficulty stems mostly from their lack of chromophores, low polarizability, and extremely weak Raman scattering, among other things, due to the inherent chemical bond nature of PFAS.^15–17^

Despite the challenges, significant progress in sensing and quantification of PFAS has been made in the development of electrochemical methods, which utilize the unique electrochemical activity and surface adsorption properties of PFAS to enable detection and quantitative analysis through changes in electrical signals.^18,19^ Similarly, semi-quantitative assays, such as the Total Oxidizable Precursor (TOP) assay and Total Organic Fluorine (TOF) analysis, have advanced PFAS detection. The TOP a say converts PFAS precursors into detectable perfluorinated compounds *via* chemical oxidation, enabling the evaluation of the total amount of latent PFAS in a sample, while TOF measures the total organic fluorine content, providing a rapid assessment of the combined concentrations of known and unknown PFAS. These methods offer a broad range of detectable PFAS species but are constrained by indirect quantification *via* redox indicators, as well as limited sensitivity (0.50 to 0.80 ng L^−1^) and selectivity.^20,21^

Raman spectroscopy is a well-known molecular-specific technique for rapid and label-free sample measurements with no or minimal requirement for sample preparation. Surface enhanced Raman scattering (SERS), in essence Raman spectroscopy aided with plasmonic structures such as Ag nanoparticles (AgNPs) for orders of magnitude enhancement of Raman signals, has enabled unprecedently leap in detection sensitivity from 10^3^ ppm *via* conventional Raman spectroscopy to below ppb and even down to single molecules. As a result, SERS is widely used for environmental analysis, food safety, drug development, and healthcare.^22–30^

SERS has been explored for PFAS detection, but most existing methods are limited by their reliance on dry samples, indirect detection using dye molecules, or modified SERS with functional groups, which restrict their broader applicability.^16,31–35^ Bai's group developed a superstructure array for Raman quantitative analysis of PFAS mixtures, using crystal violet as an indicator, achieving detection at the ppb level.^36^ Similarly, Park's group successfully detected PFOA using a self-assembled *p*-phenylenediamine nanoparticle complex, with a limit of detection (LOD) of 1.28 ppM.^37^ Despite these advancements, documented studies suggest that SERS alone may not fully leverage the advantages of Raman scattering for label-free identification and quantification of PFAS at environmentally relevant concentrations.

In addition to enhancing Raman scattering *via* robust plasmonic nanostructures, developing and employing highly sensitive methods for acquisition of weak signals offers a promising avenue for Raman measurements of PFAS. One such method is single photon detector (SPD) that converts photons to electronic signals *via* avalanche effects.^38,39^ SPD, in conjunction with the deployment of an acousto-optic tunable filter (AOTF) for dynamic wavelength selection, offers detection sensitivity superior to conventional liquid nitrogen cooled CCD Raman spectrometers, while also allowing for significant exclusion of strong background noise, including fluorescence in challenging conditions.^40–42^ Indeed, we showed in a recent study that SPD/AOTF (without AgNPs) and SERS/CCD spectrometer have comparable sensitivities in Raman measurements of trace R6G in aqueous solutions.^43^ The study was significant in that it demonstrated that SPD detection can achieve SERS-like sensitivity without Raman scattering enhancement. It further suggests that integrated SERS/SPD presents an exciting new opportunity for Raman measurements of PFAS at concentrations below ppt.

This study reports our investigation to develop and explore the combination of SERS and SPD for PFAS detection and quantification. SERS-active substrates consisting of multi-layered AgNPs deposited on Si wafer were controllably using a solution-based process. R6G was initially used as a model analyte to develop and optimize the SERS substrates for the highest possible Raman enhancement. Using the optimized substrate with 8 layers of Ag NPs, we were able to measure PFOA and PFOS at concentrations as low as 10^−15^ M. We also demonstrated the quantitative capacity of the SERS/SPD approach in concentration measurements of R6G, PFOA, and PFOS, revealing a log–scale correlation between Raman photon counts at their strongest characteristic vibrational modes and the concentrations. The *R*^2^ value is 0.98 for R6G and 0.97 for both PFOA and PFOS. The 8-layered AgNPs increased the specific surface areas and provided densely packed hotspots, creating favourable conditions for absorption and Raman signal enhancement of the target analytes. Detection of the Raman signals using SPD with time-gating for fluorescence suppression enabled sensitive measurements at high signal-to-noise ratio. This innovative SERS/SPD method has the potential to advance the frontier of sensing and measurements of PFAS, not only in technique development but also in field-deployable applications.

## 2 Experimental methods

### 2.1 Materials

Sodium citrate dihydrate (Na_3_C_6_H_5_O_7_·2H_2_O, 99%), Silver nitrate (AgNO_3_, 99%), polyallylamine hydrochloride (PAH, [CH_2_CH(CH_2_NH_2_·HCl)]_*n*_, average Mw 15 000), silicon wafer (MSE Supplies, Inc), sodium hydroxide (NaOH, 97%), Rhodamine 6G(C_28_H_31_N_2_O_3_Cl, 99%), Perfluorooctanoic acid (CF_3_(CF_2_)_6_COOH, 95%), perfluoro octane sulfonate (C_8_H_5_F_13_O_3_S, 95%).

### 2.2 Preparation of AgNPs colloidal solutions

The AgNPs solution was synthesized by the silver nitrate reduction method 44. 0.8 mL of 1% wt aqueous sodium citrate was added dropwise at a rate of 0.6 mL min^−1^ to 40 mL of 1 mM aqueous AgNO_3_ in a 50 mL beaker. The beaker was then placed in a water bath to keep the reaction temperature below 50 °C and subsequently placed in a UV chamber for 4.5 hours with continuous stirring. The final solution contained monodisperse AgNPs with an average size of 40 ± 5 nm as characterized by scanning electron microscopy (SEM; LEO 982 FEG, Carl Zeiss SMT Inc., Peabody, MA, USA). The particles exhibited a *ζ* -potential of −40 mV ± 5 (Zetasizer Nano-ZS, Malvern Instruments, Inc.) and displayed a localized surface plasmon resonance (LSPR) peak at 406 nm (Synergy HT multi-detection microplate reader, BioTek Instruments).

### 2.3 Preparation of SERS substrates with multilayered AgNPs

A 10 mm × 10 mm fixed-size silicon wafer with a thin native negatively charged oxide layer was selected as the substrate. Since the AgNPs also possess a negative surface charge, a layer of positively charged PAH was introduced as an anchoring layer. Firstly, silicon substrates were immersed in a PAH solution (0.2 mg mL^−1^, pH 9) for 20 minutes, then gently rinsed with Milli-Q water. The PAH-functionalized silicon substrates were then immersed in 2 mL of AgNPs colloidal solution (10^16^ particles/mL) and kept in the dark for 8 hours. Subsequently, the substrates with one-layer AgNPs were carefully rinsed in Milli-Q water at pH 4.5. Repeating these steps yields a multilayer AgNPs substrate.

### 2.4 SERS measurement *via* singe photo detection

**Fig. 1:**
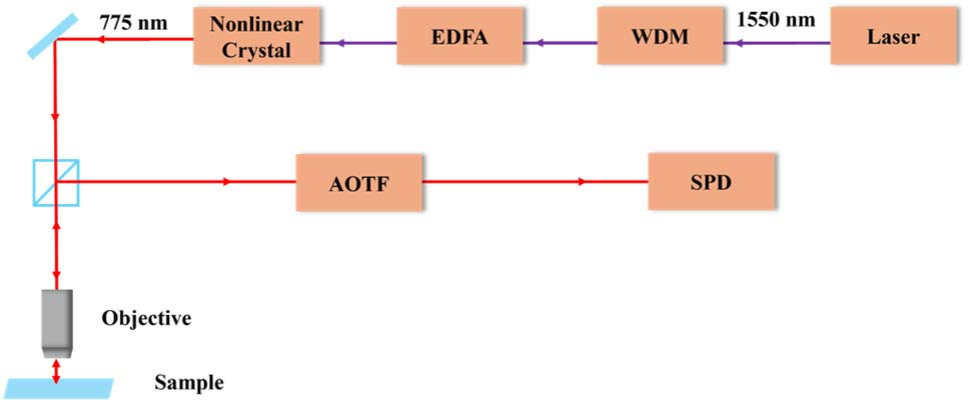
The sketch of the experiment setup. A mode-locked laser operating at a wavelength of 1550.9 nm serves as the pump source, generating second harmonic (SH) light at ∼ 775 nm. An acoustic-optic tunable filter (AOTF)-based selective single-photon detector (SPD) detects the resulting Raman output signal.

The Raman spectra obtained by a SERS and an SPD system were reported in our previous work. Briefly, the Raman signal is filtered through an AOTF to isolate signals at specific Raman shifts, which are then detected using a single-photon level detector. The 1550.9 nm pulse laser (CALMAR Laser) was selected by Wavelength Division Multiplexing (WDM), amplified by an Erbium-Doped Fiber Amplifier (EDFA), and modulated by various optical components. The Nonlinear Crystal converted the wavelength to a 775 nm pulse laser beam, then propagated through free space towards the objective lens (100×, 0.8 NA, Nikon CFI 60 LU PLANEPI ELWD) and focused on samples. The reflected light passes through a dichroic mirror, a notch filter, and the AOTF (Brimrose Corp., Tellurium Dioxide Noncollinear crystal, TEAF3-750-950). The spectrum collected by the multimode fibre-coupled SPD (Excelitas, SPCM-CD-3346-H) with a dark count of ∼ 50 Hz, a dead time of ∼ 25 ns, and a timing resolution of ∼ 350 ps. The details of this setup are shown in Fig. 1.

SERS spectra of R6G, PFOA, and PFOS at various concentrations were acquired using three independently prepared Si substrates for each concentration. Raman measurements were performed at an excitation wavelength of 1550 nm, with the laser power maintained at approximately 10 mW. For each substrate, five random spots were measured, and the spectra were averaged to obtain the final signal.

## 3 Results and discussion

### 3.1 Characterization of the SERS substrates

**Fig. 2:**
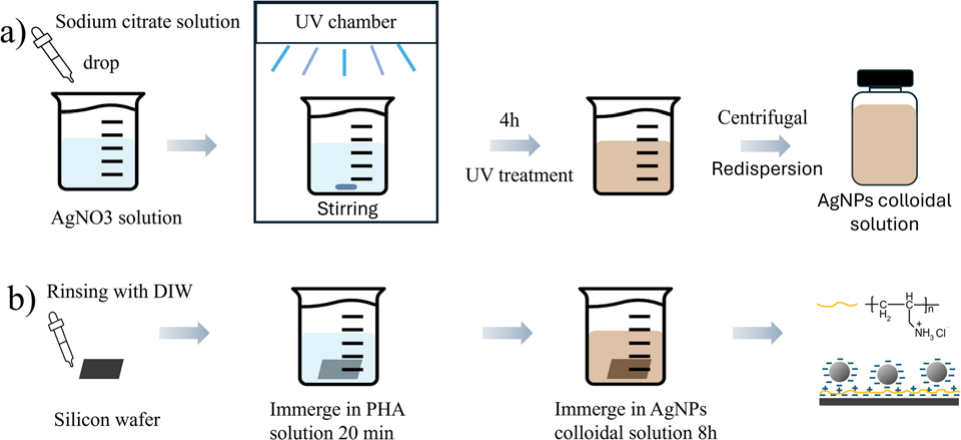
Preparation of 3D SERS active substrates and their structural characterization based on AgNPs. (a) Schematic illustration of the synthesis of AgNPs. (b) Preparation of SERS substrates with multilayered AgNPs by electrostatic attraction.

**Fig. 3:**
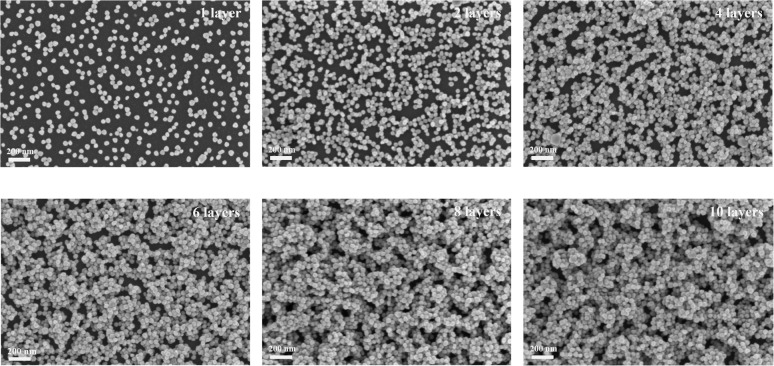
SEM images of the *n*-layer AgNPs film (*n* = 1, 2, 4, 6, 8, 10).

Colloidal AgNPs solution was synthesized using the silver nitrate reduction method,^44^ as shown in Fig. 2a and stored in a refrigerator for preservation. The AgNPs were then deposited layer by layer onto Si wafer substrate through electrostatic attraction, as illustrated in Fig. 2b. The substrates with multi-layered AgNPs were characterized using a scanning electron microscope (SEM), as shown in Fig. 3,which displays SEM images of AgNPs with varying numbers of layers (1, 2, 4, 6, 8, and 10).

The monolayered AgNPs exhibit a high degree of uniformity, with most AgNPs well-dispersed and only minimal clustering, leaving substantial areas of the Si substrate uncovered. This limited coverage restricts the formation of intense electromagnetic hotspots, which are essential for effective SERS enhancement. When the AgNPs layers increase to two, the particle coverage improves, reducing exposed substrate areas and generating more junctions (hotspots) of AgNPs, thereby facilitating SERS enhancement. As the number of layers increases to four and six, nanoparticle coverage becomes more extensive, resulting in larger clusters and a higher density of hotspots, which significantly amplify Raman signals. By the eighth layer, the AgNPs form densely and uniformly packed clusters that nearly fully cover the Si substrate, maximizing the active SERS sites. This dense nanoparticle network provides optimal conditions for electromagnetic field localization, achieving the strongest Raman signal enhancement. However, with the addition of ten layers, the uniformity of the surface deteriorates as nanoparticles aggregate into larger clusters due to oversaturation. This excessive aggregation weakens the efficiency of plasmonic coupling, increases scattering losses, and reduces the effectiveness of hotspots. Furthermore, the signals generated by the lower layers are partially attenuated or blocked by the upper layers, diminishing their contribution to the overall SERS signal. These observations reveal that an eight-layer configuration achieves the optimal balance between surface coverage and nanoparticle interactions, maximizing SERS signal enhancement. This structure effectively optimizes hotspot density without introducing adverse effects, such as excessive nanoparticle aggregation, inefficient plasmonic coupling, or attenuation of signals from lower layers.

### 3.2 Assessment of sensitivity enhancement and quantification capacity of the SERS substrates using R6G

**Fig. 4:**
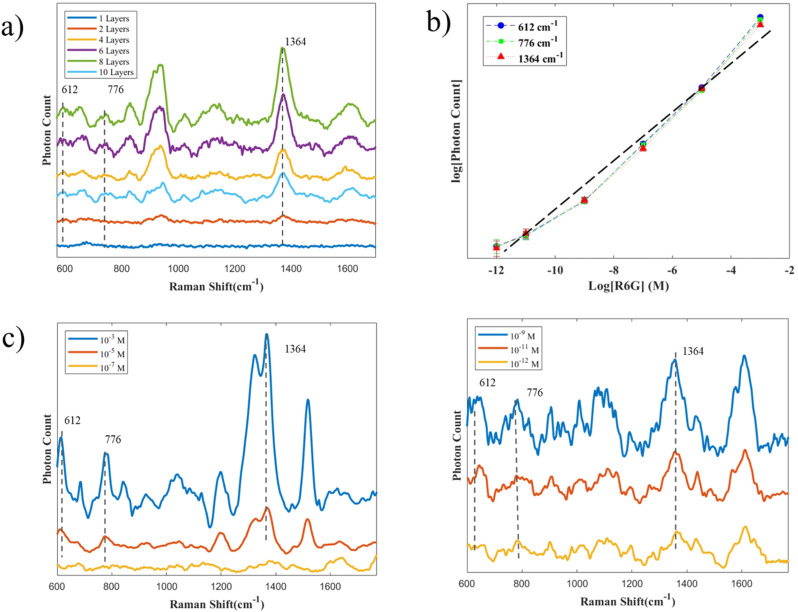
Sensitivity and reproducibility of 3D AgNPs substrate in SERS (a) Raman spectra of the *n*-layer AgNPs substrate (*n* = 1, 2, 4, 6, 8, 10). (b) The corresponding relationship between Raman intensity and detected concentrations. (c) SERS spectra were obtained from the 8-layer structure from different concentrations of R6G.

R6G is widely employed as a benchmark probe molecule in Raman spectroscopy and SERS studies due to its well-characterized spectral features and robust fluorescence emission. In particular, R6G possesses multiple characteristic Raman peaks (Table 1), which facilitate a comprehensive assessment of substrate performance. In this study, we selected R6G to systematically investigate how the number of Ag nanoparticle AgNPs layers influences the SERS performance of our AgNPs@Si substrates. As shown in Fig. 4, the SERS spectra of 10^−10^ M R6G were recorded on substrates with varying AgNPs layer numbers. At this concentration, although certain characteristic peaks were not observed, the R6G Raman peaks at 613, 774, and 1364 cm^−1^ remained detectable on our substrates. Under the same experimental conditions, as the number of AgNPs layers increased, the intensities of representative Raman peaks at 612, 776, and 1364 cm^−1^ grew, reaching a maximum at eight layers before exhibiting a marked decline. This trend is consistent with previous reports, in which SERS intensity initially improves with increasing nanoparticle layer thickness up to an optimal point, beyond which further increases lead to diminished enhancement.^45,46^

**Table 1:** Assignment of characteristic peaks in the Raman spectrum of R6G

Raman shift (cm^−1^)	Assignment
613	C–C–C ring in-plane bending
774	C–H out-of-plane bending
1188	C–H in-plane bending
1364	Aromatic C–C stretching
1510	Aromatic C–C stretching
1653	Aromatic C–C stretching

To quantitatively characterize these observations, based on the concept of SERS enhancement factor (EF), we defined the sensitivity factor (SF) for SPD. At the same exposure time and laser power, EF is calculated using the equation:1
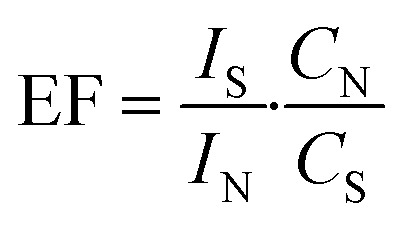
where *I*_S_ and *I*_P_ are the integrated intensities of a characteristic band from SERS and normal Raman. The parameters *C*_N_, *C*_S_ are their respective concentrations of the model analyte. Similarly, the SF is calculated using the modified equation:2
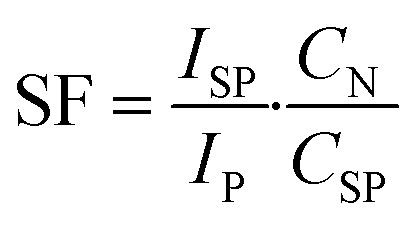
where *I*_SP_ is the integrated intensity of a characteristic band from SPD and *C*_SP_ is the concentration of the R6G analyte.

The results of SF for each multilayer configuration are summarized in Table 2. For 1–2 layers, the SF values were on the order of 10^5^, reflecting limited hotspot density. Increasing to 4–6 layers improved the SF to ∼10^6^, consistent with more extensive coverage and stronger plasmonic coupling. The 8-layer substrate reached the highest SF values (∼1.3 × 10^7^), attributable to densely distributed hotspots with enhanced local electromagnetic fields and sufficient accessible surface area for R6G adsorption. In contrast, the 10-layer substrate showed a decrease in SF to ∼5 × 10^6^, caused by reduced uniformity and hindered molecular access to deeper hotspot regions, primarily due to electromagnetic shielding and excessive scattering introduced by the additional nanoparticle layers. Consequently, increasing the number of layers beyond the optimal configuration compromises the overall enhancement effect, underscoring the importance of balancing nanoparticle density and hotspot accessibility to realize maximal SERS performance.

**Table 2:** SF of SPD with *n*-layer AgNPs substrate for R6fl peaks

Peak (cm^−1^)	612	776	1364
1 Layer	2.39 × 10^5^	1.94 × 10^5^	2.13 × 10^5^
2 Layers	3.84 × 10^5^	3.28 × 10^5^	3.72 × 10^5^
4 Layers	1.04 × 10^6^	9.69 × 10^5^	1.01 × 10^6^
6 Layers	8.99 × 10^6^	8.04 × 10^6^	8.11 × 10^6^
8 Layers	1.34 × 10^7^	1.32 × 10^7^	1.29 × 10^7^
10 Layers	5.36 × 10^6^	5.41 × 10^6^	5.25 × 10^6^

Building upon our previous qualitative assessments of the substrate's SERS performance, we further evaluated its quantitative analytical capabilities by establishing the relationship between Raman intensity and analyte concentration. We conducted five repeated experiments using R6G solutions ranging from high concentration (10^−3^ M) to trace amounts (10^−12^ M), as shown in Fig. 4c. Importantly, the Raman intensities at the three selected peaks exhibit a broad linear relationship with concentration on a logarithmic scale for both axes, as shown in Fig. 4b. Notably, at the lowest concentration, we observed significant errors and the greatest deviation from the linear trend, partly due to the poor signal-to-noise ratio and the high background noise of the photon detector at low concentrations. Overall, the *R*^2^ value of the linear fit is greater than 0.98, indicating that the substrate has strong potential for rapid quantitative detection.

**Fig. 5:**
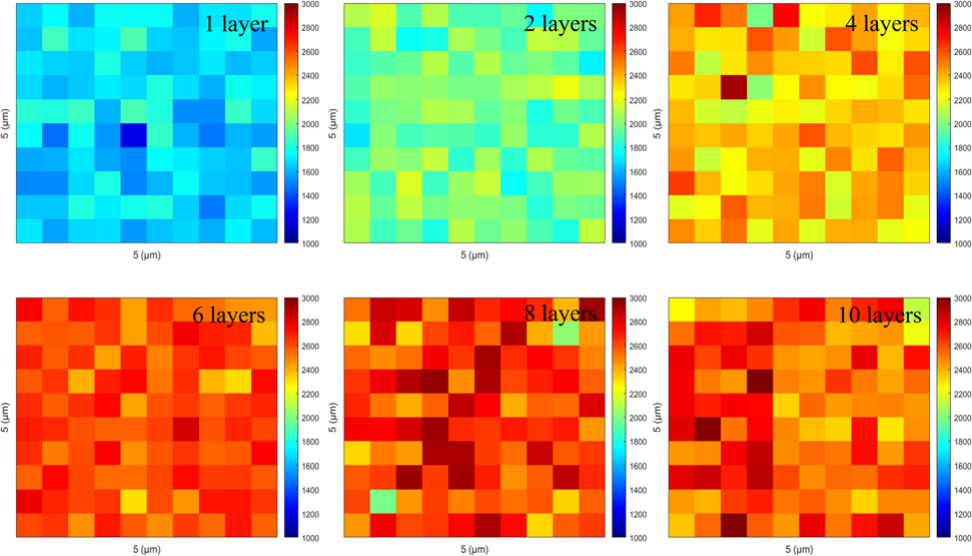
Raman mapping image of the *n*-layer AgNPs substrate (*n* = 1, 2, 4, 6, 8, 10).

To further elucidate the spatial distribution of hotspots and correlate them with the spectral results, we conducted SERS mapping experiments at characteristic Raman peaks of 10^−10^ M R6G. Mapping was performed with a step size of 0.5 μm and acquired data at each point for 0.1 s. Mapping was conducted at the peak of 1364 cm^−1^ (Fig. 5). The spatial SERS mapping clearly illustrates the evolution of hotspot distribution with increasing AgNPs layers. At low coverage (1–2 layers), only sparse and weakly enhanced regions are visible. With 4 and 6 layers, the maps reveal progressively stronger and more uniformly distributed hotspots. The 8-layer substrate exhibits the most homogeneous and intense Raman signal distribution across the scanned area, consistent with the optimal enhancement observed in the spectra. In contrast, the 10-layer substrate, while featuring some intense hotspots, showed a non-uniform spatial arrangement, leading to a decrease in overall signal intensity. The trend also been proved at 776 cm^−1^ and 1200 cm^−1^(off-peak). This indicates that simply adding more layers does not guarantee improved performance: non-uniform hotspot formation and signal shielding can outweigh any gains in hotspot quantity. The spectral measurements and the Raman mapping results confirm that the 8-layer substrate achieves the best balance between hotspot density, uniformity, and SERS enhancement.

### 3.3 SERS detection of PFAS

**Fig. 6:**
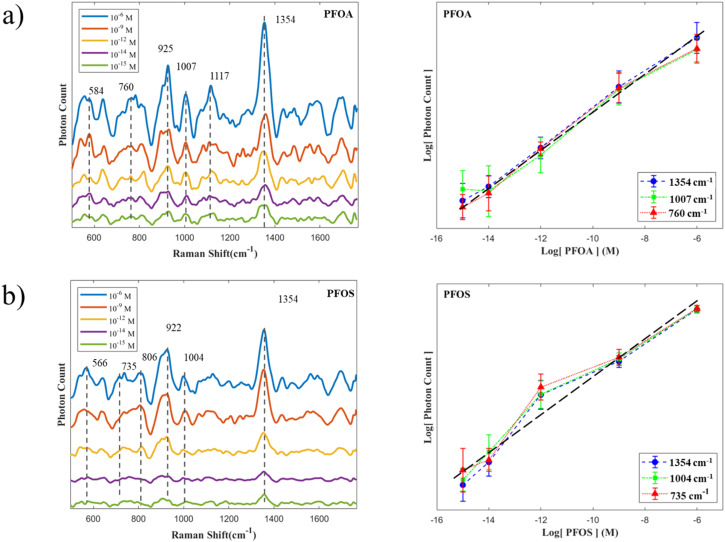
SERS spectra obtained from (a) PFOA and (b) PFOS and the corresponding relationship between Raman intensity and detected concentrations.

Further, we extended the use of the 3D structure substrate to detect commonly produced PFOA and PFOS separately, which have linear structures consisting of a hydrophobic *n*-octyl tail with a carboxylate and sulfonate head group. The PFOA and PFOS were dissolved to various concentrations (10^−6^ to 10^−15^ M) with DI water, then 1 μL of the solutions was drop-cast onto the SERS substrate. The SERS spectra are shown in Fig. 6. The spectra corresponds to different concentrations of PFAS, and the concentration calibration curves at three characteristic peaks from PFOA and PFOS respectively. The concentration dependent SERS spectra of PFOA (Fig. 6a) show that the peak around 584 cm^−1^ is indicative of *γ*(O–H) and *γ*(CF_2_), the peak around 760 cm^−1^ corresponds to *γ*(COOH), *γ*(O–H) and *β*(CF_2_), and the peaks around 1117 cm^−1^*γ*(C–C), *β*(CF_2_), and *ρ*(O–H)and 1350 cm^−1^ are associated with *β* (COOH), and *ρ*(O–H). For PFOS, (Fig. 6b) shows that the peak around 566 cm^−1^ corresponds to *γ*(SO_3_H) and *γ*(CF_2_), 735 cm^−1^ corresponds to *γ*(CF_2_), and the peaks around 806 cm^−1^ corresponds to *γ*(SO_3_H), *γ*(C–C) and *ρ*(O–H), and 922 cm^−1^ corresponds to *γ*(SO_3_H) and *ρ*(O–H), and 1004 cm^−1^ corresponds to *γ*(C–C) and *β* (CF_2_).^31,36,47–50^ During Raman measurements, some unknown peaks might be caused by Surface Enhanced Raman Scattering (SERS), which can significantly amplify certain vibrational modes that may be weak or undetectable in conventional Raman spectroscopy. Furthermore, using an SPD instead of a traditional Raman spectrometer might enhance this effect. Importantly, the achieved detection limit of 10^−15^ M corresponds to sub-ppt concentrations, which are comparable to or even surpass the sensitivity typically reported by state-of-the-art LC-MS/MS methods, highlighting the competitiveness of our approach.^12,51^

We established the relationship between Raman intensity and the concentrations of PFOA and PFOS by plotting both *x* and *y* axes on a logarithmic scale, as shown in Fig. 6. The three characteristic peaks exhibit similar trends and broadly follow linear relationships, with *R*^2^ values exceeding 0.97 for both PFOA and PFOS. Although the Raman effect for PFAS is weaker compared to R6G, resulting in higher measurement errors, the overall error remains within an acceptable range of 5%. Notably, the highest error occurs at the lowest concentration (10^−15^ M), attributable to elevated background noise from the photon detector under low-concentration conditions. Establishing this concentration calibration curve is essential for enhancing the method's applicability to detect PFAS, enabling accurate quantification across diverse matrices. This capability is particularly critical for real-world samples such as wastewater, contaminated soil, and biological tissues, where minerals, humic substances, and other coexisting contaminants may interfere with SERS responses; addressing these matrix effects through substrate modification and chemometric approaches will be the focus of our future work. Compared with conventional label-free Raman approaches, which are generally limited by the intrinsically weak Raman cross-sections of PFAS, and LC-MS/MS methods that achieve ppt-level sensitivity but require extensive sample preparation and costly instrumentation, our integrated SERS/SPD platform attains sub-ppt sensitivity by combining optimized electromagnetic hotspots with photon-counting detection for superior noise suppression.

## 4 Conclusions

In conclusion, our study developed a simple and robust method for preparing 3D AgNPs-based SERS substrates, combined with the SPD Raman measurement system, achieving enhanced sensitivity. By using R6G as a standard analyte, the 8-layer structure was validated as the most efficient configuration, providing significant SERS signal enhancements due to increased hotspot density. The strong correlation between Raman intensity and R6G concentration underscores the substrate's potential for precise quantitative analysis. Furthermore, this approach enables the detection of PFOA and PFOS at a concentration as low as 10^−15^ M and allows for the construction of corresponding concentration calibration curves. These results demonstrate that our advanced, rapid, and cost-effective detection method enables the efficient detection of PFAS. Building on these promising results, future work should focus on expanding the substrate's applicability to a broader range of analytes, including other persistent organic pollutants and biomolecules. Additionally, Coupling the SERS-SPD system with advanced data processing techniques, such as machine learning algorithms, could significantly improve the system's capacity to detect and quantify complex mixtures of pollutants in various environmental matrices. This could lead to developing comprehensive monitoring platforms for real-time detection of harmful chemicals in water, soil, and air. Moreover, exploring other types of nanomaterials and hybrid structures may yield even greater sensitivity and specificity, broadening the potential applications of this technology. Future studies will also systematically investigate the longevity of the AgNPs-coated substrates and the operational stability of the single-photon detector under repeated use, as these factors are critical for advancing the system toward reliable field applications.

## Author contributions

Tianhang Huo: conceptualization, methodology, analysis, data curation, software, writing – original draft. Yehong Li: conceptualization, methodology, software, writing – review & editing. Santosh Kumar: conceptualization, methodology, project administration. Silvana Andreescu: conceptualization, resources, writing – review & editing. Henry Du: conceptualization, methodology, supervision, writing – review & editing. Yu-Ping Huang: conceptualization, resources, writing – review & editing.

## Conflicts of interest

The authors declare no potential conflict of interest.

## Data Availability

Data supporting this study are available from the corresponding author upon reasonable request.
